# A magnetic nanobead-based bioassay provides sensitive detection of single- and biplex bacterial DNA using a portable AC susceptometer

**DOI:** 10.1002/biot.201300348

**Published:** 2013-12-19

**Authors:** Mattias Strömberg, Teresa Zardán Gómez de la Torre, Mats Nilsson, Peter Svedlindh, Maria Strømme

**Affiliations:** 1 Department of Engineering Sciences, Division of Solid State Physics, Uppsala UniversityUppsala, Sweden; 2 Department of Engineering Sciences, Division of Nanotechnology and Functional Materials, Uppsala UniversityUppsala, Sweden; 3 Science for Life Laboratory, Department of Biochemistry and Biophysics, Stockholm UniversitySolna, Sweden

**Keywords:** AC susceptometer, Brownian relaxation, Padlock probes, Probe-tagged magnetic beads, Rolling circle amplification

## Abstract

Bioassays relying on magnetic read-out using probe-tagged magnetic nanobeads are potential platforms for low-cost biodiagnostic devices for pathogen detection. For optimal assay performance it is crucial to apply an easy, efficient and robust bead-probe conjugation protocol. In this paper, sensitive (1.5 pM) singleplex detection of bacterial DNA sequences is demonstrated in a portable AC susceptometer by a magnetic nanobead-based bioassay principle; the volume-amplified magnetic nanobead detection assay (VAM-NDA). Two bead sizes, 100 and 250 nm, are investigated along with a highly efficient, rapid, robust, and stable conjugation chemistry relying on the avidin–biotin interaction for bead-probe attachment. Avidin-biotin conjugation gives easy control of the number of detection probes per bead; thus allowing for systematic investigation of the impact of varying the detection probe surface coverage upon bead immobilization in rolling circle amplified DNA-coils. The existence of an optimal surface coverage is discussed. Biplex VAM-NDA detection is for the first time demonstrated in the susceptometer: Semi-quantitative results are obtained and it is concluded that the concentration of DNA-coils in the incubation volume is of crucial importance for target quantification. The present findings bring the development of commercial biodiagnostic devices relying on the VAM–NDA further towards implementation in point-of-care and outpatient settings.

## 1 Introduction

Novel biodiagnostic principles for rapid, sensitive and cost-efficient detection of various types of biomolecules such as proteins, enzymes, and DNA, in particular DNA oligomers having sequences specific for pathogenic bacteria and viruses, are increasingly needed in today’s society [1–7]. A bioassay principle basically involves the steps of target recognition, amplification (e.g., enzymatic), labeling and read-out. Biodetection methods relying on the use of magnetic nanoparticle labels (here referred to as beads or nanobeads) combined with a read-out based on measuring changes in magnetic properties of the sample, either static or dynamic, offer unique advantages regarding cost-effectiveness and simplicity. For instance, sample preparation procedures are relatively simple while requiring low sample amounts and the read-out equipment can be made to low cost. Additionally, magnetic beads exhibit high physical and chemical stability and are relatively inexpensive to manufacture [2, 8, 9].

A vast variety of magnetic biosensor principles has so far been reported in the scientific literature [10]. Many of these operate by conjugating the surface of a magnetic sensor chip, e.g., a giant magnetoresistance (GMR) sensor [11–13], giant magnetoimpedance (GMI) sensor [14, 15], spin-valve sensor [16] or micro-Hall device [17, 18], with biomolecular probes having affinity for the target molecules. In presence of target, magnetic beads functionalized with a second type of probe also having affinity for the target bind to the sensor surface and the stray field from the beads is detected by the sensor. Another category, sometimes referred to as lab-on-a-bead, instead is based on a strategy relying on changes in the frequency-dependent (dynamic) response of the magnetic beads upon changes in the bead hydrodynamic size caused by a probe-target binding reaction occurring on the surface of the beads. To this belongs the Brownian relaxation biosensor principle theoretically outlined in 2001 by Connolly and St Pierre [19] and later on experimentally demonstrated in a protein bioassay [20].

In 2008 we published a first proof-of-concept of a novel lab-on-a-bead type magnetic bioassay principle, the volume-amplified magnetic nanobead detection assay (VAM-NDA), aiming at being suitable for rapid, simple, and cost-efficient biosensor devices to be used in point-of-care and outpatient settings [21]. Briefly, when detecting single-stranded DNA-target sequences, for instance corresponding to different types of pathogenic bacteria/viruses, the VAM-NDA relies on the steps of (i) target recognition through hybridization to a padlock probe [22–24] followed by ligation of the circular probe-target complex (DNA-circle); (ii) enzymatic amplification of the DNA-circles by rolling circle amplification (RCA) [25–28] giving a random-coiled single-stranded DNA structure (DNA-coil) [29] with a repeating sequence (the complement of the padlock probe sequence) and (iii) magnetic labeling of the DNA-coils by means of adding magnetic nanobeads exhibiting Brownian relaxation behavior and equipped with single-stranded DNA-probes (detection oligonucleotides) complementary to the DNA-coil sequence. In the last step a certain portion of the beads, determined by the DNA-coil concentration, hybridize to the DNA-coils (bead immobilization) and immobilized beads thereby undergo a dramatic hydrodynamic size increase which in turn strongly alters their Brownian relaxation frequency. By recording the frequency-dependent (complex) magnetic susceptibility of the sample, *χ* = *χ‘* – *iχ“*, target quantification can be obtained by considering the amplitude of the Brownian relaxation peak for non-immobilized nanobeads; the high-frequency peak (HFP) level or equivalently *χ“*_max_. In fact, the HFP level decreases with increasing concentration of DNA-coils. Details on the underlying magnetic theory can be found elsewhere [21].

After having used an expensive, bulky, and cryogenic liquid requiring superconducting quantum interference device (SQUID) set-up for VAM-NDA read-out in the earliest works [21, 30–33] an important step forward in the development of the VAM-NDA assay toward low-cost diagnostics was achieved by performing the read-out in a portable AC susceptometer operating at ambient temperature [34–36]. By this, the analysis time, i.e., the time needed for magnetic read-out, was reduced from around 2 h to about 30 min. The limit of detection (LOD), ∼4 pM, was found to be comparable to that of the SQUID set-up [34]. A covalent conjugation chemistry for attaching detection probes to the magnetic beads relying on the use of a cross-linker reagent (sulfo-SMCC) was employed [37]. The conjugation protocol involved a multi-step procedure requiring rather long time (around a day) and exhibiting modest oligonucleotide coupling yield (at best in the order of a few %).

In the present work, the assay is developed further toward the realization of a commercially attractive bioanalytical device. In comparison to previous work, we are now employing and optimizing a considerably more efficient (coupling yield in the order of several tens of %), much less time demanding (around 30 min of execution time), robust (oligonucleotides per bead scales linearly with added oligonucleotide amount) and stable (low loss of oligonucleotides from beads after several months of storage) detection probe conjugation scheme relying on the avidin–biotin interaction. Important to point out is also that this conjugation protocol offers the possibility to easily control the number of detection probes per bead in the low surface coverage region. From a commercial biosensor perspective such conjugation chemistry is attractive since it provides easiness and savings in time and cost (in this case low waste of oligonucleotides). Moreover, for two different nanobead sizes, 100 and 250 nm, the conjugation protocol is optimized by varying the number of detection probes attached to beads in order to minimize the singleplex LOD for a *Vibrio cholerae* (VC) target sequence. We also provide a first-time proof-of-concept of biplex detection of VC and *Escherichia coli* (EC) targets in the portable AC susceptometer. The systematic variation of the number of detection probes per bead and the biplex detection investigation also gives further insight in the bead-coil physico-chemical interaction.

## 2 Materials and methods

Sequences of targets, padlock probes, and detection oligonucleotides used in the singleplex (VC) and biplex (VC, EC) experiments can be found in Supporting information, Table S1.

### 2.1 Avidin–biotin conjugation of detection probes to magnetic nanobeads for singleplex detection

A series of 200 μL batches of oligonucleotide-functionalized 100 nm beads (BNF-Starch avidin, Micromod, Germany) with varying oligonucleotide excess with respect to the total number of beads (15-, 30-, 45-, and 60-fold excess of oligonucleotides) was prepared according to the following protocol: 40 μl of magnetic bead suspension (10 mg/mL of solid content, 6 × 10^12^ beads/mL) was placed in an Eppendorf tube. The beads were washed with 1× Wtw buffer (10 mM Tris–HCl [Sigma–Aldrich, USA], 5 mM EDTA [Sigma–Aldrich], 0.1% Tween20 [Sigma–Aldrich], 0.1 M NaCl [Sigma–Aldrich]) two times using a magnetic separation stand and re-suspended in 50 μl of 1 × Wtw buffer. 6.2, 12.5, 18.7, and 24.9 μl of 1 μM VC detection oligonucleotide solution (Biomers, Germany) was added to the magnetic beads (15- to 60-fold excess of oligonucleotides) followed by vortexing the mixture and incubation for 15 min at room temperature with rotation of the tube. Thereafter the beads were washed with 1 × PBS pH 7.5 (Sigma–Aldrich) two times, re-suspended in 200 μL of PBS and finally transferred into a new Eppendorf tube and stored in fridge at 4°C.

A series of batches of oligonucleotide-conjugated 250 nm beads (nanomag-D avidin, Micromod) with varying oligonucleotide excess with respect to the total number of beads (40-, 60- and 100-fold excess) was prepared following the same protocol as for the 100 nm beads except for starting with 200 μL of magnetic bead suspension (10 mg/mL of solid content, 4.9 ×10^11^ beads/mL) and adding 6.5, 9.75, and 16.25 μL of 1 μM VC detection oligonucleotide solution to the magnetic beads (40-, 60-, and 100-fold excess of oligonucleotides).

The average number of oligonucleotides per bead (oligonucleotide surface coverage, see Supporting information, Fig. S1) was estimated by comparing the fluorescence of the oligonucleotide-coupled magnetic nanobeads to a dilution series containing free oligonucleotides and non-functionalized beads [21]. The emission scans were performed using a fluorometer (Infinite® 200, Tecan, Sweden).

Few things should be noted concerning the selected solid content of beads (∼ 2 mg/mL and ∼ 10 mg/mL for the 100 and 250 nm bead batches, respectively) and added amounts of detection oligonucleotides in the conjugation procedure. The magnetic signal per mg for the 100 nm beads was about 4 times higher than for the 250 nm beads. To get a large enough magnetic susceptibility signal in the AC susceptometer system for the 250 nm bead samples (see further details in Section 2.5), a solid content of 10 mg/mL in the batches of conjugated beads had to be used. Consequently, about 4 times lower bead content could be used in the 100 nm bead batches. From a detection sensitivity point-of-view, as low bead concentration as possible is desirable [30]. Excesses of oligonucleotides were chosen in order to achieve similar average numbers of oligonucleotides per bead for the series of 100 and 250 nm bead batches. Moreover, since the oligonucleotide coupling yield for the 250 nm beads turned out to be lower than for the 100 nm beads (see Supporting information, Fig. S1 for details), higher oligonucleotide excesses were chosen for the 250 nm bead batches.

The oligonucleotide coupling yield was analyzed for a series of 100 nm bead batches (60-fold excess of oligonucleotides) directly after conjugation and 3 months later in order to investigate the stability of the avidin–biotin chemistry. The batches were either stored at room temperature or 4°C during these 3 months.

### 2.2 Avidin–biotin conjugation of detection probes to magnetic nanobeads for biplex detection

EC and VC detection probes were conjugated to 100 and 250 nm beads using a 30- and 60-fold excess of oligonucleotides (found to give optimal VC singleplex detection sensitivity), respectively. The conjugation protocol was as described in the previous section except for starting with 200 μL of 100 nm bead suspension (final batch volume 200 μL) and with 500 μL of 250 nm bead suspension (final batch volume 500 μL) and adding 6.25 μL of 10 μM EC detection oligonucleotide solution to the 100 nm beads and 2.44 μL of 10 μM VC detection oligonucleotide solution to the 250 nm beads. The biplex detection batch was thereafter prepared by mixing 496 μL of 250 nm bead batch and 134 μL of 100 nm bead batch.

### 2.3 Padlock probe target recognition, ligation, and rolling circle amplification for singleplex detection (VC target)

For synthesis of a 50 μL batch of 4 nM DNA-coils (1 h RCA) the following protocol was applied: A 50 μL VC ligation mix (20 nM) was prepared by mixing 5 μL of 10 × Φ29buffer (without DTT), 2.5 μL of ATP (20 mM, Thermo Fisher Scientific, USA), 1 μL of phosphorylated VC padlock probe (1 μM, Biomers, Germany), 3 μL of VC target (1 μM, DNA Technology, Denmark), 1 μL of T4 DNA ligase (1 U/μL, Thermo Fisher Scientific) and 37.5 μL of MQ water followed by incubation at 37°C during 15 min.

A 30 μL RCA mix was prepared by mixing 10 μL of VC ligation mix, 3 μL of 10 ×Φ29buffer, 2 μL of dNTP (2.5 mM, Thermo Fisher Scientific), 3 μL of BSA (2 μg/μL, sterile filtered, NEB, USA), 0.2 μL of F29 DNA polymerase (Thermo Fisher Scientific) and 11.8 μL of MQ water followed by incubation at 37°C during 60 min (RCA reaction occurring) and 65°C during 5 min (inactivation of enzymes).

Hybridization buffer was prepared by mixing 2 μL of Tris–HCl (1 M, pH 8.0, Sigma–Aldrich), 4 μL of EDTA (0.5 M, Sigma–Aldrich), 1 μL Tween-20 (10%, Sigma–Aldrich), 10 μL NaCl (5 M, Sigma–Aldrich) and 3 μL of MQ water followed by diluting with MQ water by a factor of 10. Twenty microliters of hybridization buffer was thereafter added to the RCA mix to obtain the final batch of DNA-coils.

Dilution series of DNA-coils were prepared by stepwise dilution of the 4 nM DNA-coil batch with hybridization buffer.

### 2.4 Padlock probe target recognition, ligation and rolling circle amplification for biplex detection (VC and EC targets)

Twelve different DNA-coil solutions containing different amounts of VC and EC DNA-coils (see Supporting information, Table S2) were prepared according to the following protocol: VC and EC ligation mixes (200 μL, 20 nM) were prepared separately according to the procedure in the previous section (scaled up 4 times, target and padlock sequences according to Supporting information, Table S1). Small amounts of 2 nM and 200 pM ligation mixes were prepared by diluting 20 nM mix with MQ water.

A series of 12 RCA mixes were prepared by mixing desired amounts of VC and EC ligation mixes, 16.4 μL of master mix (6 μL 10 × Φ29 buffer, 4 μL dNTP (2.5 mM), 6 μL BSA (2 μg/μL, sterile filtered) and 0.4 μL of Φ29 polymerase) and MQ water; see Supporting information, Table S2. The RCA mixes were thereafter incubated at 37°C during 60 min and 65°C during 5 min.

Final DNA-coil solutions (100 μL total volume per sample) were obtained by adding 40 μL of hybridization buffer, except for the sample having 4860 pM of both VC and EC DNA-coils. To this, due to the large volumes of ligation mixes needed, a special 35 μL mixture consisting of 4 μL Tris–HCl (pH 8, 1 M), 8 μL EDTA (0.5 M), 2 μL of Tween-20 (10%), 20 μL NaCl (5 M) and 1 μL of MQ water was added in order to obtain the same concentration of buffer salts as in the other DNA-coil solutions.

### 2.5 Dynamic magnetic measurements on samples containing DNA-coils and probe-tagged beads

Fifteen microliters of oligonucleotide-tagged nanobead batch solution was gently mixed with 15 μL of DNA-coil solution of variable concentration or hybridization buffer in case of negative control. The solution was incubated for 20 min at 55°C (this was found to be the optimal sample incubation temperature; data not shown here) and thereafter diluted with 170 μL of a 50–50 buffer mixture (50 v/v% of 1×PBS pH 7.4 and 50 v/v% of hybridization buffer). Measurements of the frequency-dependent magnetic volume susceptibility at 24°C was performed in a DynoMag®-instrument (Acreo, Sweden, frequency range 1 Hz to 250 kHz and AC field amplitude 0.5 mT). To account for slight differences in nanobead content between samples, volume susceptibility data was normalized using the constant high-frequency *χ‘* level, *χ‘*_inf_ [34]. The measured frequency dependent susceptibility for a reference sample consisting of 50–50 buffer was used to perform a background subtraction of the susceptibility data recorded for samples containing magnetic beads.

## 3 Results and discussion

### 3.1 Optimization of singleplex VAM-NDA detection sensitivity

**Figure 1:**
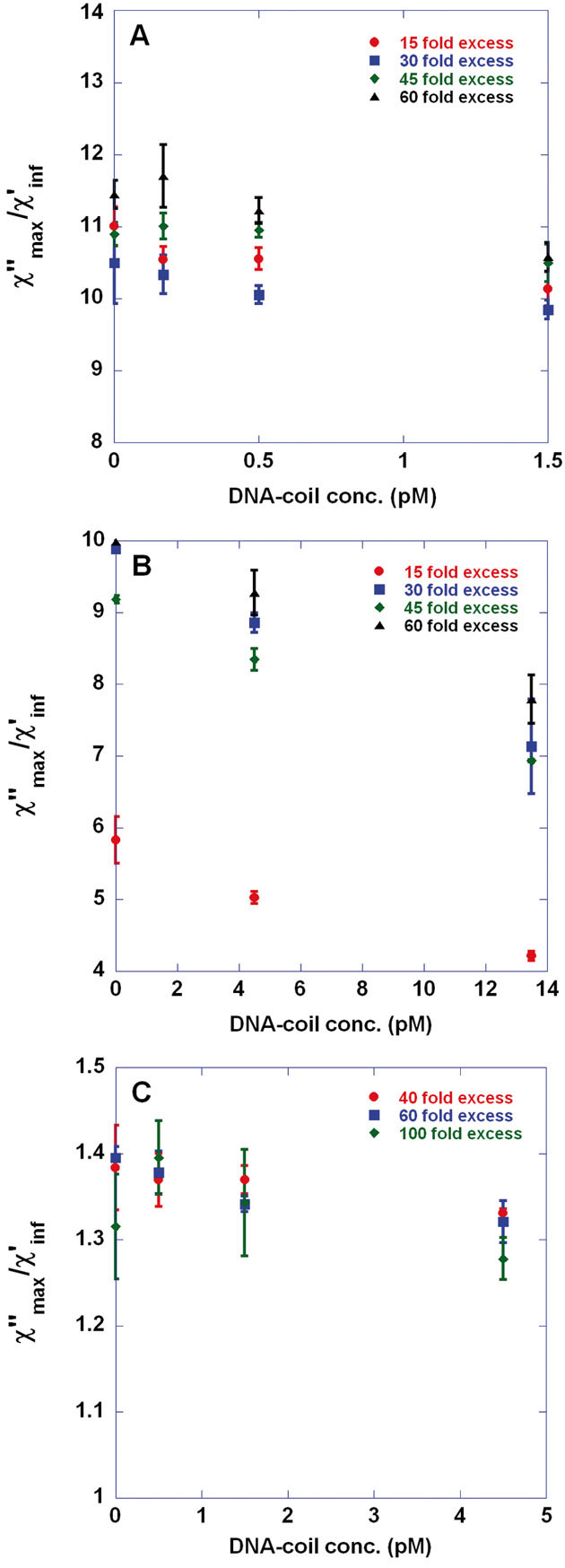
Singleplex detection of *V. cholerae* (VC) target-DNA by means of applying the volume-amplified magnetic nanobead detection assay using 100 and 250 nm magnetic nanobeads. Biodetection procedure comprised the steps of (i) target recognition through padlock probe assay giving DNA-circles; (ii) 1 h rolling circle amplification of the DNA-circles giving DNA-coils and (iii) immobilization of detection oligonucleotide-tagged magnetic nanobeads exhibiting Brownian relaxation behavior in the DNA-coils. Detection oligonucleotides were conjugated to the beads using avidin-biotin chemistry. By recording the frequency-dependent magnetic susceptibility of the sample, *χ* = *χ‘* – *iχ“*, using a portable AC susceptometer (DynoMag®) target quantification could be obtained by considering the amplitude of the Brownian relaxation peak for non-immobilized nanobeads; *χ“*_max_. (A, B) *χ“*_max_ versus VC DNA-coil concentration (0–1.5 and 0–13.5 pM in panels A and B, respectively) recorded at 24°C for 100 nm beads conjugated with 15-, 30-, 45-, and 60-fold excess of detection oligonucleotides with respect to the number of beads. (C) *χ“*_max_ versus VC DNA-coil concentration (0–4.5 pM) recorded at 24°C for 250 nm beads conjugated with 40-, 60-, and 100-fold excess of detection oligonucleotides with respect to the number of beads. To account for differences in nanobead content between samples, the *χ“*_max_ data have been normalized using the constant high-frequency *χ‘* level, *χ‘*_inf_. The error bars show one standard deviation based on three independent measurements.

Figure 1A and B show *χ“*_max_/*χ‘*_inf_ versus VC DNA-coil concentration in two different concentration ranges; 0–1.5 and 0–13.5 pM, respectively, for 100 nm beads prepared varying the excess of detection oligonucleotides with respect to the total number of beads. As can be seen from Fig. 1A, the average values for *χ“*_max_/*χ‘*_inf_ obtained at 1.5 pM coil concentration are lower than the corresponding values for the negative controls. However, the LOD is determined to 4.5 pM, Fig. 1B, defining the LOD as the lowest DNA-coil concentration, *c*, that is statistically different from the negative control; *χ“*_max_ (0 pM)/*χ‘*_inf_ (0 pM) – 3SD(0 pM) > *χ“*_max_ (c)/*χ‘*_inf_ (c) + 1SD(c). This LOD is obtained for beads prepared using a 20- and 30-fold excess of oligonucleotides.

Optimal bead immobilization can be defined by two criteria; (i) lowest LOD and (ii) highest percentage of immobilized beads for a given DNA-coil concentration. The latter equals 1 – [*χ“*_max_ (c)/*χ‘*_inf_ (c)]/[*χ“*_max_ (0 pM)/*χ‘*_inf_ (0 pM)]. For 30- and 45-fold oligonucleotide excess and for 13.5 pM of DNA-coils, 28 and 25% of the beads are immobilized, respectively. Based on the two criteria above beads prepared by a 30-fold excess of oligonucleotides give optimal bead immobilization.

Figure 1C presents *χ“*_max_/*χ‘*_inf_ versus VC DNA-coil concentration (0–4.5 pM) for 250 nm beads conjugated with 40-, 60-, and 100-fold excess of detection oligonucleotides. Following the same argumentation regarding optimal immobilization, 250 nm beads prepared with 60-fold excess of oligonucleotides perform optimally. According to the above definition, the lowest LOD is found to be 1.5 pM, which is an improvement compared to our previously reported measurements on 250 nm beads (LOD 4 pM, ∼1800 detection probes per bead, sulfo-SMCC conjugation chemistry) of nanomag-D type [34].

Oligonucleotide surface coverage versus VC oligonucleotide excess is shown for 100 nm and 250 nm beads in Supporting information, Fig. S1. As can be seen, the avidin–biotin conjugation chemistry performs very robustly for the 100 nm beads since the surface coverage scales linearly with the oligonucleotide excess and with very small batch-to-batch variations. Also, the coupling yield is very high; ∼70%. For the 250 nm beads a linear trend is also observed but with much larger spread and somewhat lower coupling yield; ∼40–50%.

The stability of the oligonucleotide attachment to the 100 nm beads is presented in Supporting information, Table S3. One can see that ∼6 and 20% of the oligonucleotides had detached from the bead surface when the beads were stored at 4°C and at room temperature, respectively.

### 3.2 Investigation of biplex VAM-NDA detection possibilities

Multiplexed VAM-NDA detection of target-DNA sequences, for instance corresponding to several types of pathogenic bacteria or viruses, can be accomplished in two ways; (i) singleplex detection of one analyte (corresponding to one type of DNA-coil) at the time, possibly in several parallel channels, (ii) simultaneous detection of several analytes (corresponding to several types of DNA-coils, i.e., with different repeating sequences) within the same sample volume by using several bead sizes (mixed with each other in suitable proportions) where each size is equipped with a detection probe hybridizing to only one type of DNA-coil. The latter methodology has been described earlier [31] where quantitative biplex detection and qualitative triplex detection of bacterial target sequences were demonstrated using a SQUID system for read-out. In the first case 130 nm beads in combination with 250 nm beads (both of nanomag-NH_2_ type) was used while for the latter one more bead size was added (80 nm beads of BNF-Starch type with NH_2_ surface). Detection probes were attached to the beads by covalent cross-linker chemistry, 1 h RCA was employed and DNA-coil concentrations in the range of 4–300 pM were considered.

**Figure 2:**
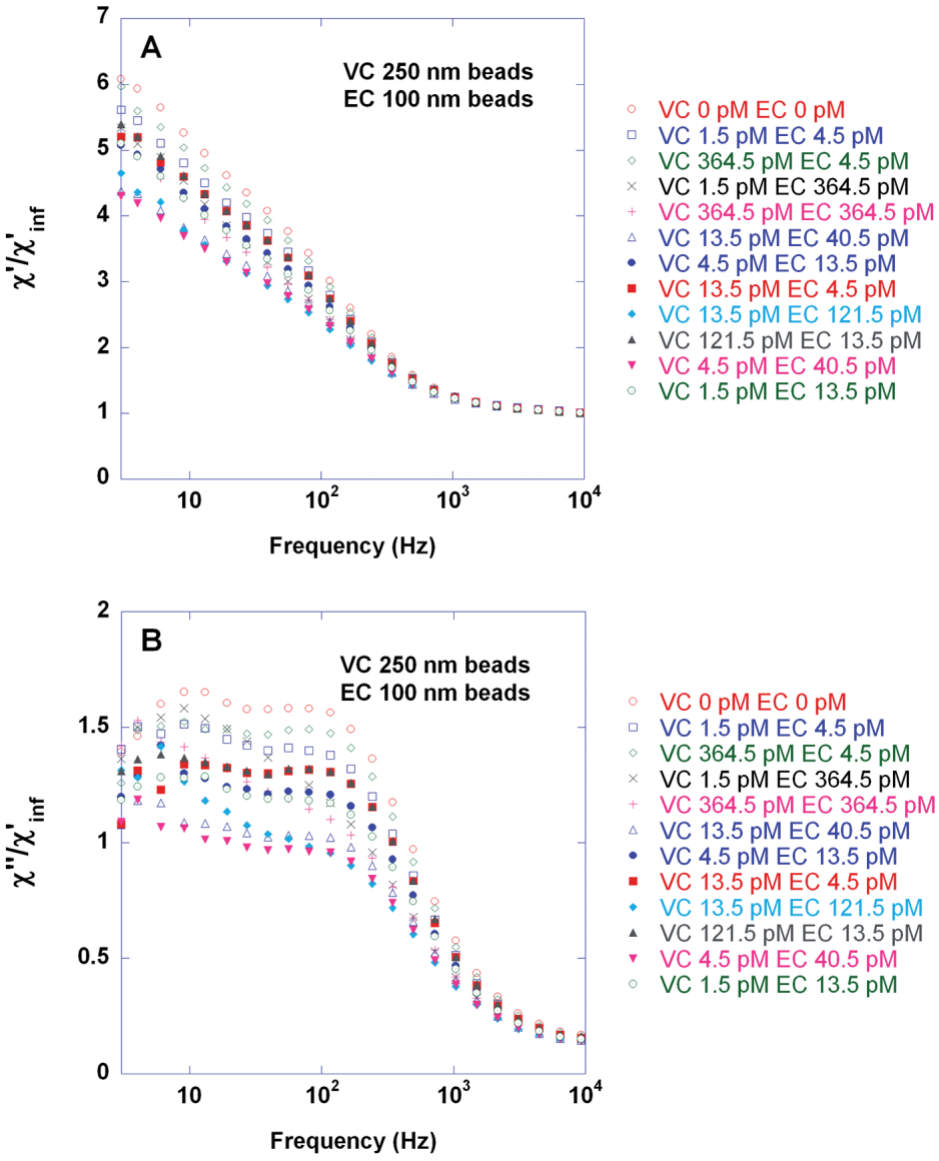
Biplex detection of *V. cholerae* (VC) and *E. coli* (EC) target-DNA for twelve combinations of target concentrations by means of the volume-amplified magnetic nanobead detection assay and using 250 nm and 100 nm magnetic nanobeads, respectively. Biodetection procedure comprised the steps of (i) target recognition performed separately for VC and EC through padlock probe assay giving DNA-circles; (ii) 1 hr rolling circle amplification of the mixtures of VC and EC DNA-circles giving a series of samples containing different combinations of concentrations of VC and EC DNA-coils and (iii) iimmobilization of a mixture of 250 nm and 100 nm magnetic nanobeads exhibiting Brownian relaxation behavior and conjugated with VC and EC detection oligonucleotides using avidin-biotin chemistry. EC and VC detection probes were conjugated to 100 nm and 250 nm beads using a 30 and 60 fold excess of oligonucleotides (found to give optimal VC singleplex detection sensitivity), respectively. Target quantification could be obtained by recording the frequency-dependent magnetic susceptibility of the sample, *χ* = *χ‘* – *iχ“*, using a portable AC susceptometer (DynoMag®) followed by fitting each *χ“* curve to a double Cole–Cole expression (sum of two Cole–Cole distributions) in order to extract *χ“*_max_ (HFP level) for VC and EC. The figure shows real (A) and imaginary (B) parts of the magnetic susceptibility recorded at 24°C and normalized using the constant high-frequency *χ‘* level, *χ‘*_inf_. Each concentration combination was measured once.

**Figure 3:**
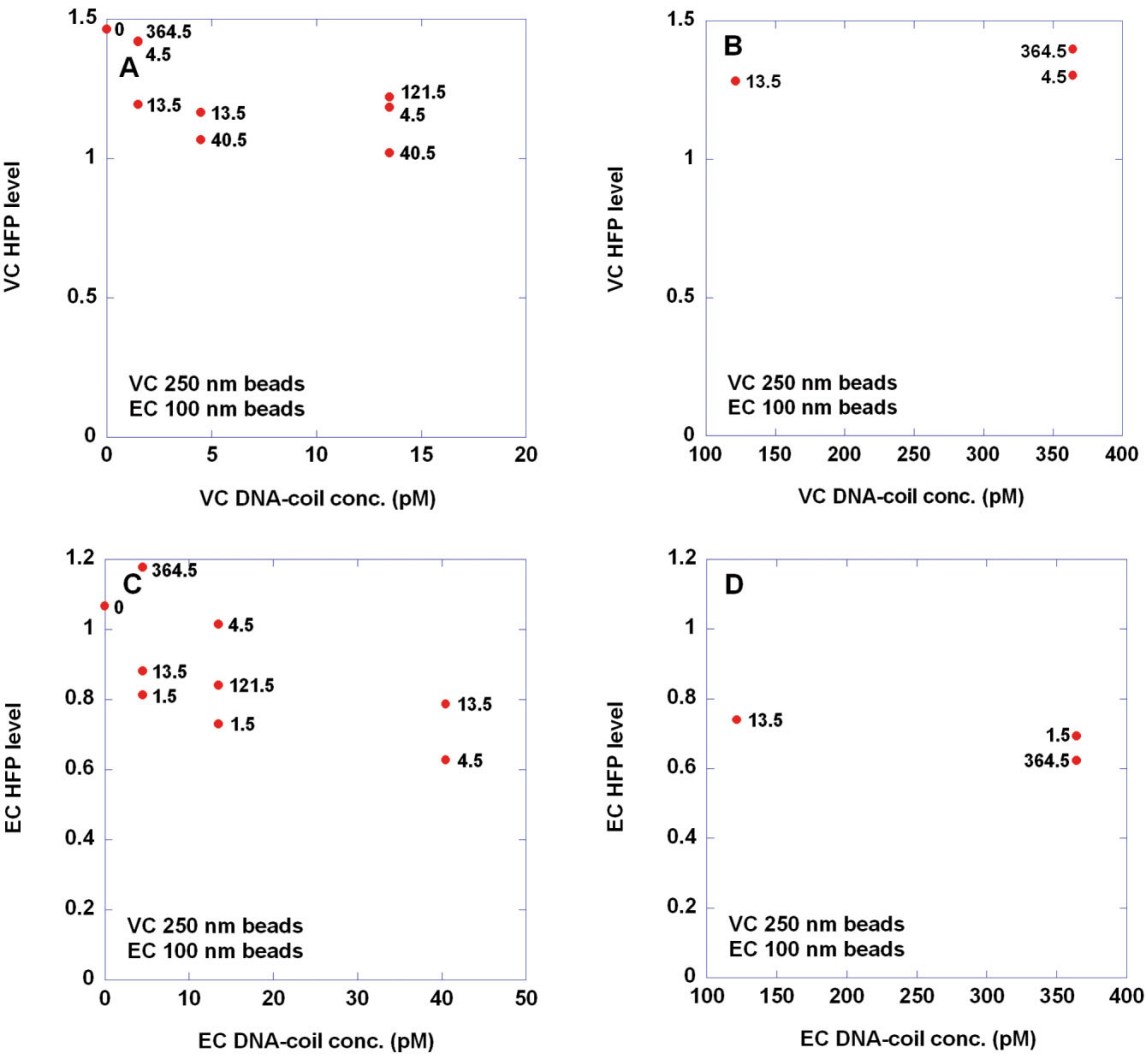
Extraction of HFP levels for *V. cholerae* (VC) and *E. coli* (EC) target in the biplexing study. Twelve combinations of DNA-coil concentrations were considered, each of them measured once (for details, see the caption of Fig. 2). The figure shows VC (A, B) and EC (C, D) HFP levels extracted from fitting the curves in Fig. 2B (each corresponding to one particular DNA-coil concentration combination) to a double Cole–Cole expression (sum of two Cole–Cole distributions). Details on the double Cole–Cole curve fitting analysis can be found elsewhere [31]. The DNA-coil concentration of the opposite target is indicated in each case by bold numbers appearing close to each dot.

Here we evaluate the possibility to perform biplex detection of VC and EC target sequences in the DynoMag portable AC susceptometer using the bead types considered above for singleplex detection and conjugated to achieve optimal VC singleplex detection sensitivity. Twelve combinations of target concentrations within similar ranges as in the previous study [31] were considered. Real- and imaginary parts of the normalized magnetic susceptibility for these samples are shown in panels A and B, respectively, of Fig. 2. Note that due to dilution of the samples with 50–50 buffer after incubation the DNA-coil concentration in the incubation volume (30 μl) is a factor of 6.7 times higher than in the final measurement volume (200 μl); cf. Supporting information, Table S2. The VC and EC HFP levels extracted from fitting the curves in Fig. 2B to a double Cole–Cole expression (sum of two Cole–Cole distributions) are displayed in Fig. 3. The DNA-coil concentration of the opposite target is indicated in each case by bold numbers appearing close to each data point. Compared to the previous study [31], the scatter in the HFP level, when keeping one DNA-coil concentration constant and varying the other, is larger. Furthermore, quite surprisingly, bead immobilization occurs more efficiently for moderate DNA-coil concentrations (in the range of few tens of pM) than for the highest ones (364.5 pM). Conclusively, very high DNA-coil concentrations in the incubation volume (nM levels) pose a technical limitation. On the other hand, a semi-quantitative analysis is achievable if DNA-coil concentrations do not exceed a few tens of pM in the final measurement volume. Moreover, as indicated in Fig. 3C, it can be observed that when keeping one DNA-coil concentration constant and varying the opposite, the HFP level tends to increase upon increasing the concentration of the other type of DNA-coil. From a fundamental view point this observation is interesting since it supports the hypothesis [31] that presence of non-matching DNA-coils impedes the immobilization of beads in their matching DNA-coils, for instance by diffusing into the non-matching DNA-coils and/or by interacting with them electrostatically.

## 4 Summary and conclusion

The present work has taken the VAM-NDA closer to practical implementation for detection of bacterial target sequences in a portable AC susceptometer operating at ambient temperatures and with short analysis times. These achievements have been made possible by applying a conjugation chemistry (avidin–biotin) for attachment of detection probes to the beads considerably more efficient than previously used protocols relying on covalent cross-linker chemistries. The avidin–biotin conjugation protocol is fast (around half an hour protocol), simple (no need of cross-linker reagent, very few steps), robust (probe surface coverage almost linearly proportional to the excess of probes added with respect to total number of beads), efficient (coupling yield around 40–70% depending on bead size) and stable (6% of the oligonucleotides detaches from the 100 nm beads when stored at 4°C). From a commercial biosensor perspective such a conjugation protocol is attractive since it provides easiness and savings in time and cost (in this case low waste of oligonucleotides). By optimizing the number of detection probes per bead the singleplex detection sensitivity could be improved (detection limit down to 1.5 pM; one single enzymatic amplification step of the probe–target complexes was employed) compared to previously reported work [34]. The applied conjugation scheme also provided the opportunity to systematically investigate the impact of varying the detection probe surface coverage on the beads upon bead immobilization behavior. The finding that a certain oligonucleotide surface coverage may give optimal bead immobilization could possibly be explained in terms of that low surface coverage gives low bead-coil electrostatic repulsion but less strong bead-coil binding whereas high surface coverage gives the opposite effects.

We have also, for the first time in the portable susceptometer system, explored the possibilities to perform biplex VAM-NDA detection. A semi-quantitative analysis was obtained and it was found that very high DNA-coil concentrations in the incubation volume (nM levels) pose a technical limitation to the assay. Thus, the concentration of DNA-coils in the incubation volume is of crucial importance for the target quantification. Moreover, observations were made supporting an earlier put-forward hypothesis that presence of non-matching DNA-coils impedes the immobilization of beads in their matching DNA-coils, for instance by diffusing into the non-matching DNA-coils and/or by interacting with them electrostatically.

The present findings, thus, take the development of commercial biodiagnostic devices relying on magnetic VAM-NDA-based read-out for detection of, e.g., pathogenic bacteria and/or viruses further toward implementation in point-of-care and outpatient settings. The presented results are deemed important not only for implementation of the assay in portable AC suseptometers, but also for the possibility of developing miniaturized chip-based sensors for assay read-out [38, 39].

## Abbreviations

EC: *Escherichia coli*
HFP: high-frequency peak
LOD: limit of detection
VAM-NDA: volume-amplified magnetic nanobead detection assay
VC: *Vibrio cholerae*
