# Supplementary material for: A magnetic nanobead-based bioassay provides sensitive detection of single- and biplex bacterial DNA using a portable AC susceptometer
Source: Biotechnol J. 2013 Dec 19;9(1):137–45. doi: 10.1002/biot.201300348 (PMC3910167; doi:10.1002/biot.201300348)
Supplement: Supplementary file 1 [file biot0009-0137-sd1.pdf]

Biotechnology
Journal

Supporting Information for DOI 10.1002/biot.201300348

A magnetic nanobead-based bioassay provides sensitive detection of single- and biplex bacterial DNA using a portable AC susceptometer

Mattias Strömberg, Teresa Zardán Gómez de la Torre, Mats Nilsson, Peter Svedlindh, and Maria Strømme

© 2013 Wiley-VCH Verlag GmbH & Co. KGaA, Weinheim

Table S1. Sequences of targets, padlock probes and detection oligonucleotides used in the singleplex (VC) and biplex (VC, EC) experiments.

**Name**	**Sequence**
Vibrio cholerae target (VC)	5'-CCCTGGGCTCAACCTAGGAATCGCATTTG-3'
Padlock probe for VC	5'-TAGGTTGAGCCCAGGGACTTCTAGAGTGTACCGACCTCAGTAGCCGTGACTATCGACTTGTTGATGTCATGTGTCGCACCAAATGCGATTCC-3'
Detection oligo for VC	Biotin-5'-TTTAGTAGCCGTGACTATCGACT-3'
Escherichia coli target (EC)	5'-ACGTCGCAAGACCAAAGAGGGGGACCT-3'
Padlock probe for EC	5'-CTTTGGTCTTGCGACGTCAGTGGATAGTGTCTTACACGATTTAGAGTGTACCGACCTCAGTAGCCGTGACTATCGACTAGGTCCCCCT-3'
Detection oligo for EC	Biotin-5'-TTTTTTTTTTTTTTTTTTTTGTGGATAGTGTCTTACACGA-3'

Table S2. Preparation of RCA mixes for the biplex detection.

**VC DNA-coil conc. (pM)**	**Vol. of VC lig. mix 20 nM (µl)**	**Vol. of VC lig. mix 2 nM (µl)**	**Vol. of VC lig. mix 200 pM (µl)**	**EC DNA-coil conc. (pM)**	**Vol. of EC lig. mix 20 nM (µl)**	**Vol. of EC lig. mix 2 nM (µl)**	**Vol. of MQ water (µl)**
0	0			0	0		43.6
20			10	60		3	30.6
4860	24.3			60		3	16.3
20			10	4860	24.3		9.3
4860	24.3			4860	24.3		0
180		9		540	2.7		31.9
60		3		180		9	31.6
180		9		60		3	31.6
180		9		1620	8.1		26.5
1620	8.1			180		9	26.5
60		3		540	2.7		37.9
20			10	180		9	24.6

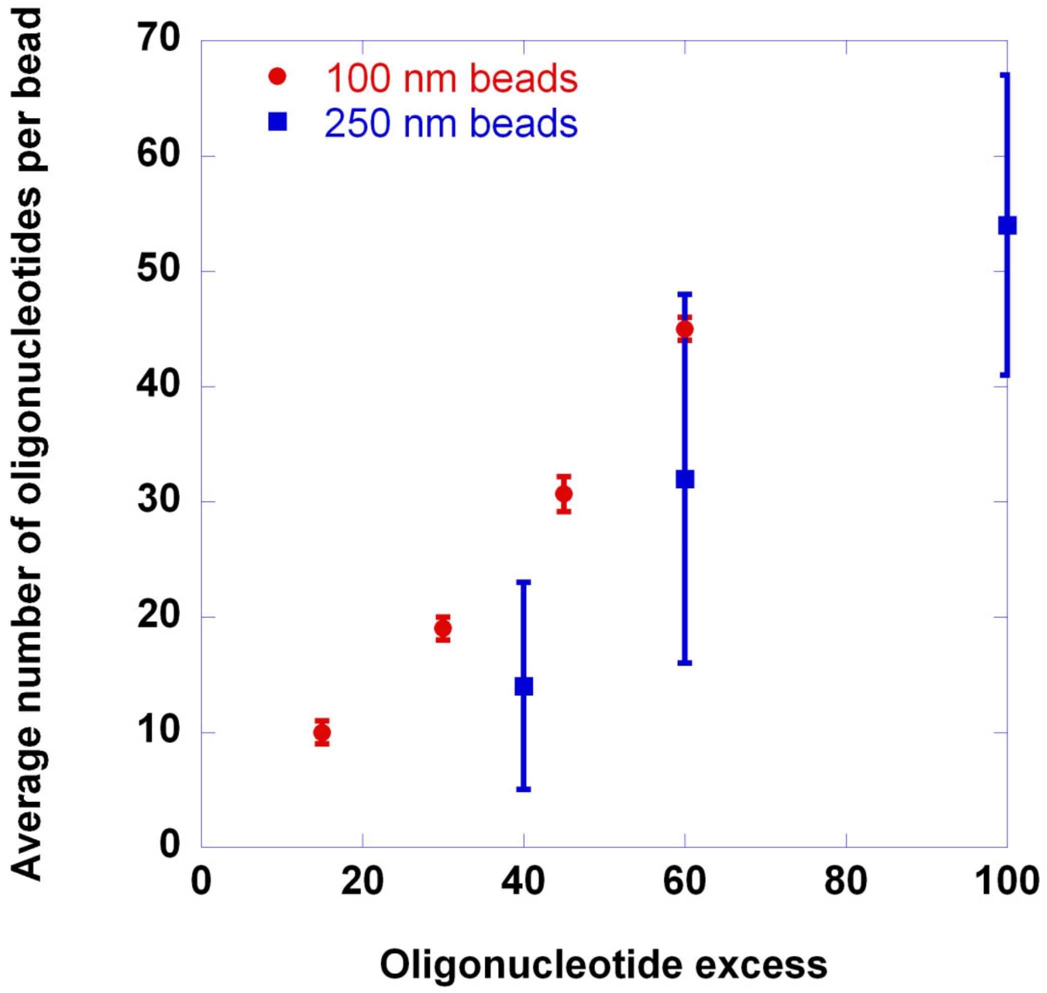

Figure S1. Average number of VC detection probes per bead (oligonucleotide surface coverage) for 100 nm and 250 nm beads vs. the excess of oligonucleotides with respect to the total number of beads used in the avidin-biotin conjugation protocol. Standard deviations are based on triplicates.

Table S3. Oligonucleotide surface coverage for 100 nm beads with 60 fold excess of oligonucleotides stored at room temperature and 4 ^{0}C. The fluorescence analysis was performed directly after conjugation and three months later. Standard deviations are based on triplicates.

**Sample storage conditions**	**Average number of oligonucleotides per bead**		**Loss (%)**
	**Direct after conjugation**	**Three months later**	
Room temperature	49 ± 4	39 ± 1	20
4^{0}C	50 ± 3	47 ± 2	6
